# Cepharanthine may inhibit the proliferation of prostate cells by blocking the EGFR/PI3K/AKT signaling pathway: comprehensive network analysis, molecular docking, and experimental evaluation

**DOI:** 10.3389/fphar.2025.1654757

**Published:** 2025-11-24

**Authors:** Yin Huang, Jingxing Bai, Biao Ran, Jinze Li, Bo Chen, Zeyu Chen, Jie Chen, Yan Wang, Jin Li, Qiang Dong, Qiang Wei, Dehong Cao, Liangren Liu

**Affiliations:** 1 Department of Urology, West China Hospital, Sichuan University, Chengdu, China; 2 Department of Urology, Chengdu University of TCM, Chengdu, China; 3 Research Core Facility, West China Hospital, Sichuan University, Chengdu, China

**Keywords:** Cepharanthine, benign prostatic hyperplasia, network analysis, moleculardocking, WPMY-1, Bph-1

## Abstract

**Introduction:**

Pharmacological studies have confirmed that Cepharanthine (CEP) can exert anti-inflammatory, antioxidant and anti-fibrotic effects. However, there is no systematic study on whether CEP targets and regulates the core pathological link of benign prostatic hyperplasia (BPH) - matrix hyperplasia.

**Methods:**

First, the CEP structure was obtained through PubChem. Combined with BPH targets from the GeneCards/OMIM/TTD database, potential targets were obtained by intersection using Venny 2.1. Then, the PPI network was constructed using STRING, and top 20 core targets were identified using Cytoscape 3.9.1. GO/KEGG enrichment analysis was performed using the DAVID database. Based on the CB-Dock platform, CEP was molecularly docked with key targets, the protein structure was derived from AlphaFold2 and PDB, and the binding energy was calculated by the VINA algorithm. Furthermore, human prostate stromal cells WPMY-1 and benign prostatic hyperplasia cells BPH-1 were used as a model. The Celigo full-field scanning system dynamically monitored proliferation from 0 to 96 h, DNA synthesis was quantified by EdU staining, and apoptosis was detected by Annexin V-APC/PI or Annexin V-FITC/PI double staining flow cytometry. Finally, the effect of CEP on the expression of key target genes was analyzed by Western blot.

**Results:**

Network analysis showed that 96 cross-targets were significantly enriched in the PI3K-AKT, MAPK and HIF-1 pathways. Molecular docking confirmed that CEP strongly bound to EGFR (−9.2 kcal/mol), AKT1 (−7.7 kcal/mol), and FN1 (−9.6 kcal/mol). *In vitro* experiments showed that CEP inhibited WPMY-1 (IC_50_ = 6.396 μM) and BPH-1 (IC_50_ = 2.355 μM) proliferation in a dose-dependent manner. Treatment of BPH-1 and WPMY-1 cells with 2.5 μM and 5 μM CEP for 48 h, respectively, significantly reduced the proportion of EdU^+^ cells in both cell lines. Celigo counting revealed a significant decrease in both cell lines after 24–96 h of CEP treatment. Flow cytometry revealed a significant increase in the total apoptotic rate of both WPMY-1 and BPH-1 cells after CEP treatment. Western blot analysis revealed that CEP inhibited EGFR and AKT phosphorylation and FN1 expression in WPMY-1 and BPH-1 cells in a dose-dependent manner.

**Conclusion:**

This study confirmed for the first time the effectiveness of CEP in targeted regulation of prostatic hyperplasia. However, the *in vivo* efficacy needs to be verified in testosterone-induced animal models in the future.

## 1 Introduction

Benign prostatic hyperplasia (BPH) is a common urinary system disease in middle-aged and elderly men. Its prevalence increases with age. About 20% of men in their 40s are affected, and up to 70% of men in their 60s are affected (Elsaqa and El Tayeb, 2024; Schally et al., 2025). It is reported that from 1990 to 2021, the global incidence of BPH has risen sharply from 6.5 million to 13.5 million per year (Schally et al., 2025). The pathological characteristics of BPH are abnormal proliferation of prostate stroma and epithelial cells, which leads to enlarged prostate volume and mechanical obstruction of the urethra, causing lower urinary tract symptoms (LUTS) such as frequent urination, urgency, and dysuria, which seriously impair the patient’s quality of life (Le Guévelou et al., 2025; Singh et al., 2025). Current clinical treatment is mainly based on drug intervention (Liedtke et al., 2024; Song et al., 2024). α1-adrenergic receptor blockers relieve symptoms by relaxing prostate smooth muscle, but are prone to orthostatic hypotension; although 5α-reductase inhibitors can reduce the size of the gland, they are accompanied by side effects such as decreased libido and erectile dysfunction (Papet et al., 2025; Soda et al., 2025). Although surgical treatment can effectively relieve obstruction, there are risks of complications such as postoperative bleeding, infection and retrograde ejaculation (Passarelli et al., 2025; Singh et al., 2025; Vanthoor et al., 2025). Therefore, the development of new targeted therapies that are both efficient and safe, especially drugs targeting the core pathological link of matrix hyperplasia, has become an urgent need for current research.

Cepharanthine (CEP) is a dibenzylisoquinoline alkaloid isolated from the root of the traditional Chinese medicine *Epistephanine*, with a chemical structure of C_37_H_38_N_2_O_6_, which has unique transmembrane transport properties and multi-target regulation capabilities (Lu et al., 2023; Xia et al., 2023). A large number of studies have confirmed that CEP can exert anti-inflammatory, antioxidant and anti-fibrotic effects by inhibiting TNF-α-mediated NF-κB activation, scavenging reactive oxygen free radicals (ROS), and blocking platelet aggregation (Bailly, 2019; Liu et al., 2022; Shi L. et al., 2023). In a pulmonary fibrosis model, CEP can inhibit fibroblast activation by regulating macrophage M2 polarization and reducing the expression of fibrosis-related factors (Bao et al., 2024). In addition, CEP has shown dual efficacy in inhibiting both viral replication and cytokine storm in the treatment of COVID-19, highlighting its potential to regulate complex signaling networks (Liu et al., 2022; Wang et al., 2023; Xia et al., 2023; Leng et al., 2024). It is worth noting that studies have shown that CEP can inhibit the ERK signaling pathway by enhancing the expression of DUSP1, thereby exerting anti-tumor effects on prostate cancer *in vitro* and *in vivo* (Dong et al., 2025). However, there is no systematic study on whether CEP can target and regulate the proliferation and apoptosis of prostate stromal cells and thus intervene in the progression of BPH.

Based on the multifactorial pathogenesis of BPH and the multi-pathway regulatory characteristics of CEP, this study first used the “network analysis combined experimental verification” strategy to analyze the molecular mechanism of CEP in the treatment of BPH: network analysis was used to screen the cross-targets of CEP and BPH, combined with molecular docking to simulate the drug-target protein interaction mode, and finally cell experiments were used to verify the regulatory effects of CEP on the proliferation, apoptosis and EGFR/PI3K/AKT/FN1 signaling axis of prostate stromal cells (WPMY-1) and benign prostatic hyperplasia cells (BPH-1). We propose a core scientific hypothesis: CEP may block the process of prostate matrix hyperplasia by inhibiting the EGFR/PI3K/AKT signaling cascade and the expression of its downstream fibronectin FN1, providing a new strategy for the targeted treatment of BPH.

## 2 Materials and methods

The various software, online database platforms, and online tools utilized in presented study were listed in Supplementary Appendix 1.

### 2.1 Drug efficacy assessment

We utilized PubMed and Web of Science to investigate the clinical applications of the Chinese botanical drug extracts CEP, which named QianJinTengSu in Chinese. Additionally, the TCMIP database (http://www.tcmip.cn/TCMIP/index.php/) was employed to examine efficacy and treatment data related to CEP. Our objective was to obtain precise preliminary insights into the underlying mechanisms by which CEP may treat BPH.

### 2.2 Collection of CEP targets

The canonical SMILES notation and molecular structure of CEP were retrieved from the PubChem database (https://pubchem.ncbi.nlm.nih.gov/, accessed on 14 November 2024). Utilizing this structural information, we identified potential drug targets by employing various databases, including SwissTargetPrediction (http://www.swisstargetprediction.ch/), ChEMBL (https://www.ebi.ac.uk/chembl/), SEA Search Server (https://sea.bkslab.org/), and STITCH (http://stitch.embl.de/). To ensure data integrity and reliability, the search was restricted to targets specific to *Homo sapiens*. Following the aggregation of results, redundant targets were filtered out, resulting in a comprehensive and refined library of potential CEP drug targets (Shi S. et al., 2023).

### 2.3 Screening of BPH-related targets

The keywords “benign prostatic hyperplasia”, “BPH”, and “hyperplasia, prostatic” were employed to retrieve BPH-related targets from three disease-specific databases: GeneCards (https://www.genecards.org/), OMIM (https://omim.org/), and TTD (https://db.idrblab.net/ttd/). The retrieved BPH-related targets were consolidated, and duplicate entries were excluded. Using the “Relevance score” in the GeneCards database as the screening basis, only genes with scores higher than the median score of all targets are retained to ensure that the screened targets have a high correlation with the disease, thereby improving the reliability of subsequent analysis.

### 2.4 Screening of key targets and construction of protein–protein interaction (PPI) network

To explore the potential targets of CEP in treating BPH, we performed a cross-analysis of CEP and BPH-related Targets and generated a Venn diagram using Venny 2.1 tools (https://bioinfogp.cnb.csic.es/tools/venny/index.html) to identify overlaps. These intersecting targets were subsequently imported into the STRING database (http://string-db.org/) to construct a PPI network. The species was limited to *Homo sapiens*, and the minimum interaction score threshold was set to 0.4, with all other parameters maintained as default. The PPI network was visualized using Cytoscape 3.9.1 software, and the degree of each target was calculated using the CytoNCA plugin. Key targets were identified based on their degree values, and molecular interactions were further analyzed.

### 2.5 GO and KEGG pathways enrichment analyses

To elucidate the biological mechanisms underlying the potential targets of CEP in the treatment of BPH, we conducted Gene Ontology (GO) and Kyoto Encyclopedia of Genes and Genomes (KEGG) pathway enrichment analyses. Utilizing the DAVID database (DAVID Functional Annotation Tools), which provides comprehensive gene function annotations across biological processes (BP), cellular components (CC), and molecular functions (MF), we identified significant GO terms and KEGG pathways. Pathway enrichment was deemed statistically significant at a false discovery rate (FDR) of less than 0.05. The top 10 GO terms and top 20 KEGG pathways were thoroughly summarized.

Furthermore, KEGG pathway enrichment analyses for the central targets of CEP in BPH treatment were performed using multiple databases, including DAVID, FUMA (https://fuma.ctglab.nl/gene2func), and Metascape (https://metascape.org/gp/index.html#/). This comprehensive approach aimed to investigate the signaling pathways and biological processes mediated by core targets in BPH treatment, thereby elucidating key mechanisms. Finally, the GO and KEGG enrichment results were visualized and interpreted using the online tool WeiShengXin (https://www.bioinformatics.com.cn/), facilitating the exploration of drug-disease signaling pathways and biological processes, and highlighting the underlying mechanisms.

### 2.6 Molecular docking of CEP with key targets

To elucidate the molecular interactions and binding modes between CEP and key target proteins, we conducted molecular docking simulations. This structure-based approach predicts receptor-ligand binding geometries and affinities. Ligand files of CEP components in SDF format were retrieved from the PubChem database (https://pubchem.ncbi.nlm.nih.gov/), and three-dimensional structural models of proteins RAC-alpha serine/threonine-protein kinase 1 (AKT1), epidermal growth factor receptor (EGFR), Proto-Oncogene Tyrosine-Protein Kinase Src (SRC), and fibronectin 1 (FN1) were sourced from AlphaFold2 (https://alphafold.com/), UniProt (https://www.uniprot.org/), and the Protein Data Bank (PDB) (https://www.rcsb.org/). Docking was performed using the CB-DOCK platform (https://cadd.labshare.cn/cb-dock2/index.php), and the resulting poses were analyzed and visualized with Discovery Studio 2019 software.

### 2.7 Chemicals and reagents

CEP was purchased from Selleck Chemicals (Houston, USA). The purity of CEP was ≥98% as determined by HPLC. Store at −20 °C in a dry place away from light. Before use, prepare a 10 mM stock solution in DMSO and aliquot to avoid repeated freeze-thaw cycles. All CEP working solutions used in experiments were freshly diluted from this stock solution. DMEM high glucose medium, fetal bovine serum, trypsin, and PBS solution was purchased from Thermo Fisher-Scientific (Massachusetts, USA). Penicillin-streptomycin solution was purchased from Merck (Darmstadt, Germany). Cell Counting Kit (CCK)-8 was purchased from Dojindo Molecular Technologies (Maryland, USA). EdU cell proliferation assay kit, Annexin V-APC/PI Apoptosis Kit and Annexin V-FITC/PI Apoptosis Detection kit were purchased from Elabscience (Wuhan, China). Antibodies against AKT (No. 75692), phosphorylated AKT (Ser473) (No. 4060), EGFR (No. 4267), phosphorylated EGFR (Tyr1068) (No. 3777), FN1 (No. A0056-3), and GAPDH (No. 2118) were purchased from Cell Signaling Technology, Inc. (Boston, USA). Anti-rabbit secondary antibody was purchased from Absin (Shanghai, China).

### 2.8 Cell culture

WPMY-1 (human prostate stromal cells) and BPH-1 (​benign prostatic hyperplasia-1) were purchased from American Type Culture Collection (ATCC) (Maryland, USA) and cultured in DMEM high-glucose medium supplemented with 10% fetal bovine serum and 1% penicillin-streptomycin double antibody solution at 37 °C in a humidified atmosphere of 5% CO_2_. The culture medium was changed every 2–3 days according to the cell growth rate and state. When the cell confluence reached about 80%, the cells were passaged in proportion. All experiments were performed using cells between passage 3 and passage 20 to ensure phenotypic stability and minimize the effects of cellular senescence.​

### 2.9 CCK-8 assay

10,000 WPMY-1 cells/well or 10,000 BPH-1 cells/well were seeded in a 96-well plate (100 μL culture medium per well) for 24 h (37 °C, 5% CO_2_) to adhere to the wall. Then, DMSO and 2.5, 5, 10, and 20 μM CEP were added for 48 h. Three replicate wells were set for each concentration, and the edge wells were filled with PBS buffer to eliminate the evaporation effect. After the intervention, 10 μL CCK-8 reagent was added to each well, gently shaken and mixed, and incubated for 2 h. The absorbance at 450 nm was detected using an ELISA reader (Biotek, Vermont, USA), and blank wells and solvent control wells were set simultaneously to correct the background. The cell viability was calculated according to the formula: (OD_450_ of the experimental group - OD_450_ of the blank group)/(OD_450_ of the DMSO control group - OD_450_ of the blank group) × 100%. The four-parameter nonlinear regression dose-effect curve was fitted with CEP concentration (X-axis) and survival rate (Y-axis), and the drug concentration at which the inhibition rate reached 50% was calculated as IC_50_. Subsequently, 10,000 WPMY-1 (or BPH-1) cells/well were inoculated and pre-cultured for 24 h using the same method, and then divided into a DMSO control group and a 5 μM CEP (for BPH-1, the CEP concentration was 2.5 μM) (CEP concentration determined according to IC_50_) treatment group (3 replicates per group). At five time points of 0, 24, 48, 72, and 96 h, 10 μL of CCK-8 reagent was directly added to each well, and the absorbance at 450 nm was detected after incubation for 2 h (the blank well background was deducted). The proliferation rate was calculated according to the formula: (treatment group OD_450_–0 h OD_450_)/(control group OD_450_–0 h OD_450_) × 100%, and the time-proliferation rate curve was drawn.

### 2.10 Celigo cell counting

A 96-well plate (final volume of 100 μL per well) inoculated with 10,000 WPMY-1 (or BPH-1) cells was divided into a DMSO control group and a 5 μM CEP (for BPH-1, the CEP concentration was 2.5 μM) (CEP concentration determined according to IC_50_) treatment group. The cells were pre-cultured for 24 h before administration to ensure stable cell adhesion. Non-labeled live cell counts were performed using a Celigo full-view cell scanning analyzer (Nexcelom Bioscience, Boston, USA) at five time points: 0, 24, 48, 72, and 96 h. The instrument was set to bright field full-well scanning mode (resolution 1 μm/pixel), and the Direct Cell Counting Application was used to perform high-speed imaging of the entire plate. The software automatically segmented the cell image and counted the number of live cells in each well. To reduce batch errors, the same culture plate was used for dynamic tracking at multiple time points throughout the process, and the culture plate was gently shaken before each scan to redistribute the suspended cells.

### 2.11 EdU staining

The culture medium of WPMY-1 (or BPH-1) cells (96-well plate, 10,000 cells/well, 100 μL culture medium/well) treated with DMSO or 5 μM CEP (for BPH-1, the CEP concentration was 2.5 μM) (CEP concentration determined according to IC_50_) for 48 h was discarded, 100 μL culture medium containing 10 μM EdU was added to each well, and incubated at 37 °C for 2 h to allow EdU to be incorporated into DNA; the EdU culture medium was discarded, the cells were washed twice with PBS, and 50 μL 4% paraformaldehyde was added to each well for fixation at room temperature for 30 min; the fixative was discarded, and 100 μL PBS containing 0.5% Triton X-100 was added for permeabilization for 10 min; the permeabilization solution was discarded, and 50 μL of Click reaction solution was prepared according to the proportion of the kit, and it was added to each well for reaction at room temperature for 30 min in the dark; the reaction solution was discarded, PBS was washed three times, and 100 μL DAPI (1 μg/mL) was added to each well for nucleus staining in the dark for 10 min; after washing with PBS, the cells were observed under a fluorescence microscope (Olympus, Tokyo, Japan), and the EdU^+^ (green) and DAPI^+^ (blue) cells were counted to calculate the proliferation rate.

### 2.12 Cell apoptosis detection

The culture medium of WPMY-1 cells (96-well plate, 10,000 cells/well) treated with DMSO or 5 μM CEP for 48 h was discarded, and the cells were gently washed once with PBS. The adherent cells were digested with EDTA-free trypsin, and the floating cells (including apoptotic cells) were combined and collected by centrifugation at 1,000×g for 5 min. The supernatant was discarded, and the cells were washed twice with pre-cooled PBS and resuspended with 195 μL 1× Binding Buffer. 100 μL of the cell suspension was taken, 5 μL Annexin V-APC and 10 μL propidium iodide (PI) staining solution were added, and the mixture was gently vortexed and incubated at room temperature in the dark for 15 min 300 μL Binding Buffer was added to terminate the staining, and the cells were detected by flow cytometry within 1 h (Beckman, California, USA). Apoptosis rate analysis: Annexin V-APC^+^/PI^−^ represents early apoptotic cells, Annexin V-APC^+^/PI^+^ represents late apoptotic/necrotic cells, and the increase in apoptosis rate was calculated using the DMSO group as the control. BPH-1 cells were seeded in 6-well plates at a density of 2 × 10^5^ cells per well and treated with CEP at concentrations of 0 and 2.5 μM for 48 h. Cells were then collected for apoptosis analysis using an Annexin V-FITC/PI Apoptosis Detection kit (Elabscience, Wuhan, China) according to the manufacturer’s protocol. Briefly, cells were resuspended in 500 μL of 1× binding buffer and stained with 5 μL Annexin V-FITC and 5 μL PI in the dark at room temperature for 15–20 min. Apoptosis was assessed using a flow cytometer.

### 2.13 Western blot

WPMY-1 cells or BPH-1 cells (6-well plate, 2 × 10^5^ cells/well) treated with DMSO, 2.5 μM and 5 μM CEP for 48 h were discarded from the culture medium, washed twice with pre-cooled PBS, and 100 μL RIPA lysis buffer was added to each well for lysis on ice for 30 min. The cells were scraped and transferred to a centrifuge tube, centrifuged at 12,000×g and 4 °C for 15 min, and the supernatant was taken. The protein concentration was determined by BCA method and adjusted to equal concentration. 20 μg protein sample was mixed with 5× loading buffer and denatured by boiling at 100 °C for 10 min. The cells were separated by 10% SDS-PAGE gel electrophoresis and transferred to PVDF membrane by wet transfer method. The cells were blocked with 5% skim milk at room temperature for 1 h, and primary antibodies were added: AKT, p-AKT, EGFR, p-EGFR, FN1, GAPDH, and incubated at 4 °C overnight. The cells were washed with TBST 3 times, and HRP-labeled secondary antibodies were added for incubation at room temperature for 1 h. After washing with TBST, ECL chemiluminescence was developed. ImageJ (NIH, USA) software was used to analyze the grayscale values ​​of the bands. The ratio of the target protein to GAPDH was used to correct the loading error. The phosphorylation level was calculated according to the ratio of p-AKT/AKT and p-EGFR/EGFR.

### 2.14 Statistical analysis

All cell experiments (including CCK-8, EdU, flow cytometry, and Western blot) were repeated three times independently. The data are expressed as mean ± standard deviation (mean ± SD). Multiple groups were compared using one-way analysis of variance (ANOVA) followed by Tukey’s post hoc test, and statistical significance was set at P < 0.05.

### 2.15 Research statement

This study adhered to the four pillars of ethnopharmacology best practices: (1) Traditional use: CEP has a history of use in traditional Chinese medicine; (2) Quality assurance: HPLC-verified high-purity standards were used; (3) Pharmacological evaluation: Multiple *in vitro* assays were used to verify biological activity; and (4) Ethical: All cell lines were commercially available, and no human or animal experiments were performed.

## 3 Result

### 3.1 Initial network assessment of CEP efficacy

The standardized name for CEP in the TCMIP database is Epistephanine, with a chemical formula of C_37_H_38_N_2_O_6_ and a drug-likeness score of 0.248. CEP is a natural alkaloid known for its inhibitory effects on TNF-α-mediated NF-κB activation, plasma membrane lipid peroxidation, platelet aggregation, and cytokine production. These properties contribute to its anti-inflammatory, antioxidant, and anti-tumor activities. Notably, in 2022, traditional Chinese medicine, including compounds like CEP, demonstrated efficacy in treating COVID-19.

Benign prostatic hyperplasia has been associated with various mechanisms, including sex hormone regulation, peptide growth factor signaling, inflammatory pathways, apoptosis, oxidative stress, and smooth muscle activity. Collectively, these findings lay a critical groundwork for subsequent systematic and comprehensive studies assessing the therapeutic potential of CEP in BPH.

### 3.2 Screening of potential targets of CEP in the treatment of BPH

**FIGURE 1:**
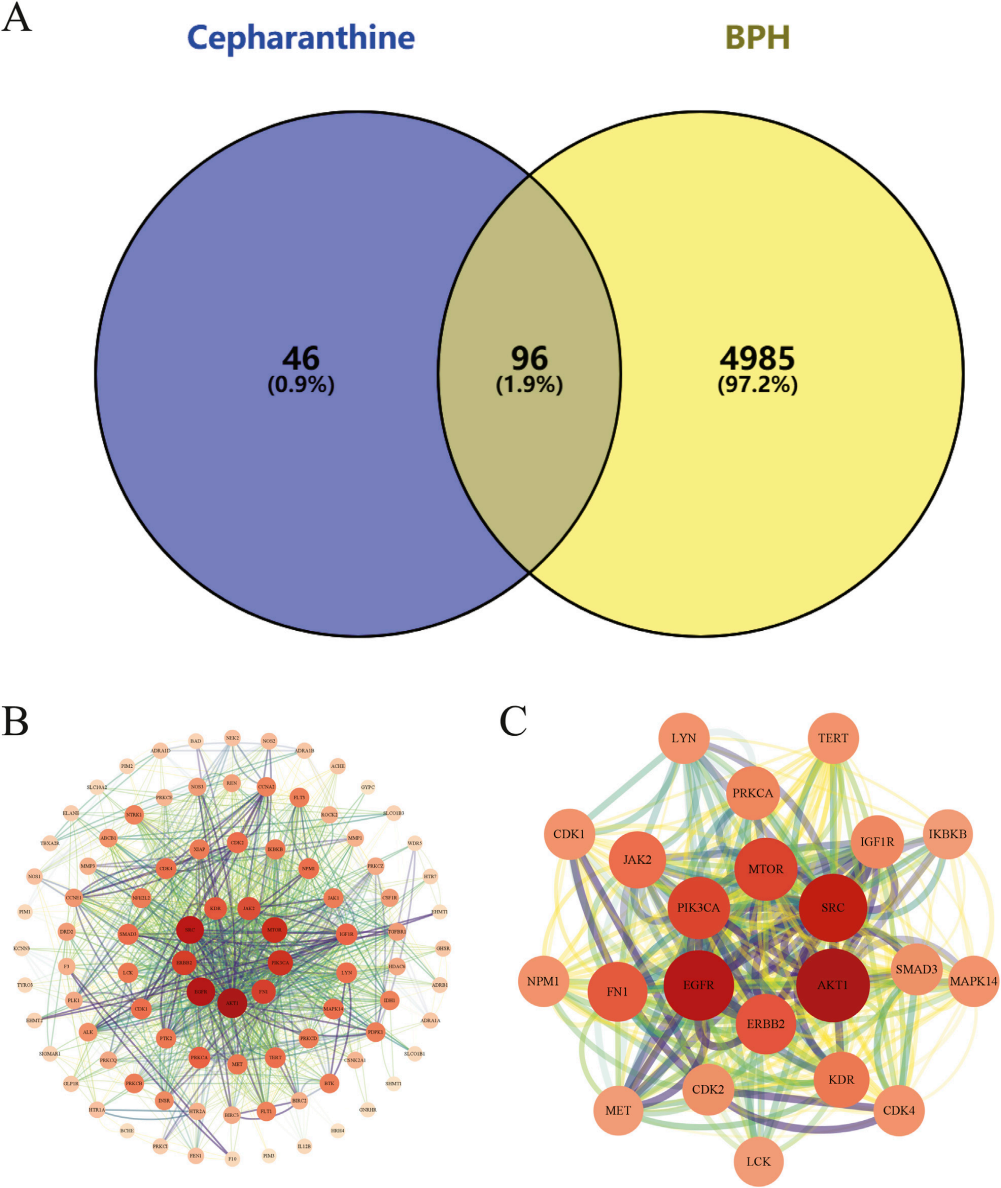
**(A)** The Venn diagram illustrates the intersection of CEP and BPH targets. Specifically, the 142 CEP targets were intersected with the 5,081 BPH-associated targets, revealing 96 potential targets that may mediate the therapeutic effects of CEP on BPH; **(B)** The PPI network of potential targets visually represents interactions between potential targets, with node size and color indicating degree values, and edge thickness and darkness reflecting connectivity scores; **(C)** The PPI network of core targets highlights the functional associations among the 20 core targets, providing insights into the molecular mechanisms by which CEP may exert therapeutic effects on BPH.

This study initially identified 142 therapeutic targets of CEP from the SwissTargetPrediction, ChEMBL, SEA Search Server, and STITCH databases, and 5,081 targets highly associated with BPH through analysis of the GeneCards, OMIM, and TTD databases. By integrating and deduplicating these target sets, 96 overlapping targets were obtained as potential targets for CEP in the treatment of BPH (Figure 1A) (Supplementary Appendix 2).

### 3.3 PPI network analysis and key target screening

We constructed a PPI network using the STRING database, comprising 96 nodes and 666 edges. The network exhibited an average node degree of 13.9, an average local clustering coefficient of 0.545, and a PPI enrichment p-value of <1.0e-16, indicating significant interactions among the targets.

The topological properties of the network nodes, including degree and betweenness centrality, were analyzed using Cytoscape software, resulting in an optimized PPI network visualization (Figure 1B). Through this analysis, 20 core targets of CEP for treating BPH were identified (Table 1). A focused PPI network was then generated (Figure 1C) to illustrate interactions among these core targets. The top 4 targets based on degree values were AKT1, EGFR, SRC, and FN1. These proteins regulate cell proliferation, cell cycle control, apoptosis, and signal transduction.

**TABLE 1:** Core targets screened from PPI network (Top20).

Gene name	Betweenness	Degree	Closeness	Eigenvector	LAC
AKT1	1548.275022	114	0.713178	0.253853	29.122807
EGFR	924.282395	104	0.676471	0.255147	32.076923
SRC	909.66478	98	0.666667	0.242159	30.44898
FN1	492.463796	72	0.597403	0.192392	27.888889
MTOR	310.876854	82	0.62585	0.227926	32.780488
PRKCA	298.081082	54	0.550898	0.142625	21.333333
PIK3CA	279.722982	80	0.613333	0.2265	34.1
JAK2	276.78629	64	0.575	0.184885	28
ERBB2	248.422122	74	0.601307	0.212281	32.432432
IGF1R	188.212553	52	0.550898	0.163992	27.384615
KDR	165.883327	60	0.571429	0.175644	27.333333
MET	164.861141	42	0.531792	0.139978	24.571429
CDK2	96.869706	48	0.516854	0.128244	20.333333
CDK1	92.895015	46	0.513966	0.124452	19.130435
CDK4	76.830621	46	0.519774	0.140985	23.478261
TERT	64.975678	46	0.531792	0.151694	27.478261
IKBKB	64.13025	42	0.525714	0.116004	19.428571
LYN	64.111675	46	0.544379	0.138012	21.913043
LCK	61.128043	42	0.525714	0.122328	21.333333
NPM1	60.786992	46	0.531792	0.143039	24.869565

### 3.4 Functional and pathways enrichment analysis of key targets

#### 3.4.1 GO and KEGG pathway mapping of potential targets

**FIGURE 2:**
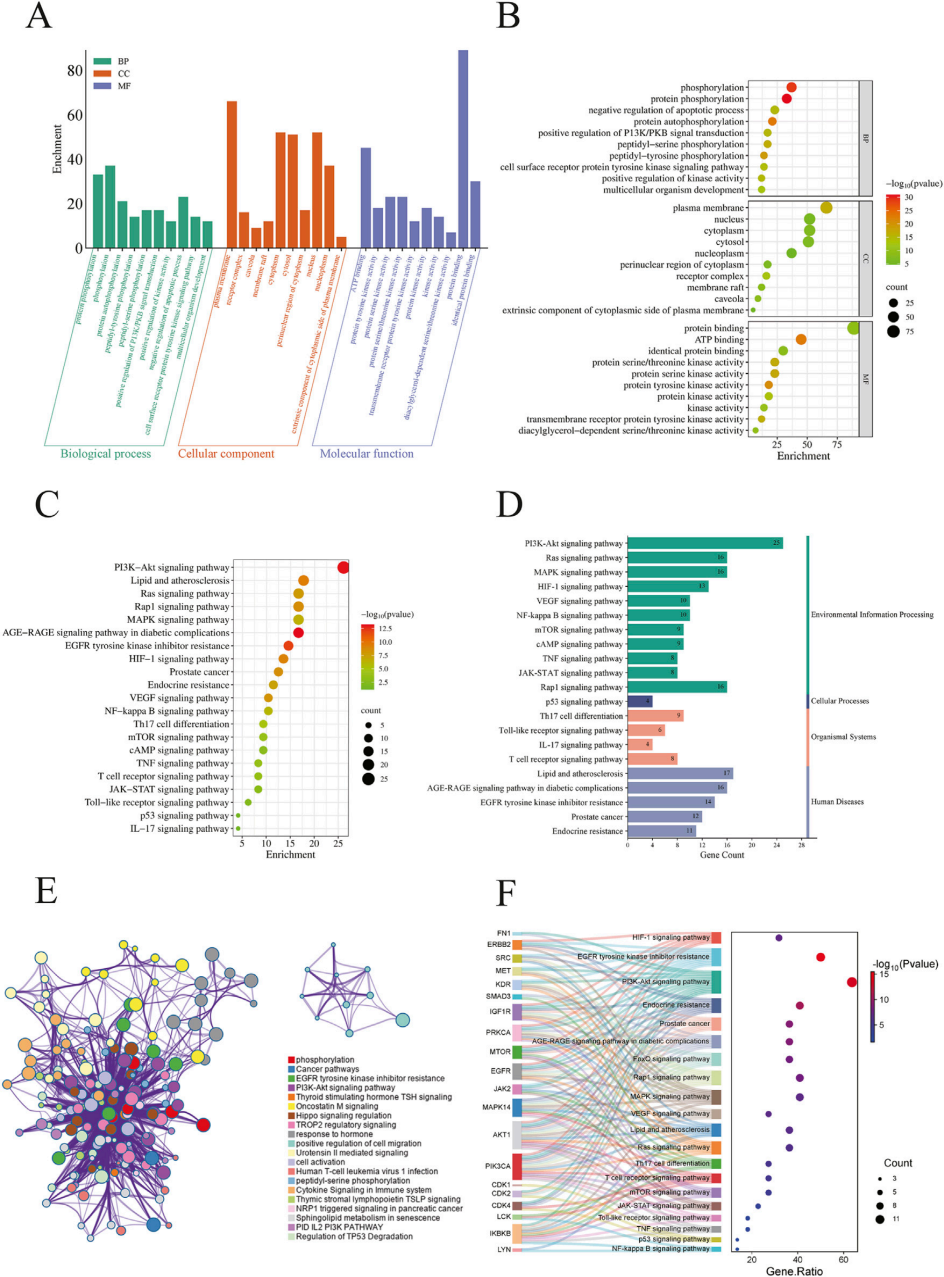
**(A)** Histogram displaying the top 10 enriched terms in each GO category, ranked by FDR value. The height of each bar represents the gene count, indicating the degree of enrichment within the category; **(B)** Bubble plot where the bubble size reflects gene expression in a specific term, and the color intensity corresponds to the FDR value—the smaller the FDR, the higher the enrichment significance; **(C)** The bubble plot shows the top 20 enriched KEGG pathways, with bubble size representing the number of enriched genes and color intensity indicating the pathway’s significance; **(D)** The histogram illustrates the enrichment frequency of each pathway, where the bar length corresponds to the gene count and the color reflects the enrichment significance; **(E)** Enriched KEGG terms were selected from the core target list based on higher enrichment levels and grouped into functional clusters. A network was constructed based on associations and similarities, with different colors representing distinct clusters; **(F)** This figure visualizes the enrichment of core target pathways, selecting 20 KEGG pathways based on comprehensive FDR. Overlapping target genes for each pathway are connected by gray lines. Bubble size represents the target count, and bubble color intensity indicates the enrichment degree.

We conducted GO and KEGG pathway analyses on 96 potential CEP targets associated with the treatment of BPH in, utilizing the DAVID database, limiting the species to *Homo sapiens*. GO analysis identified a total of 532 statistically significant terms, including 387 BP, 55 cellular components CC, and 90 MF. To prioritize the most relevant GO terms, we ranked them based on their false discovery rate (FDR) values and selected the top 10 terms with the lowest FDR, informed by previous BPH studies, for visualization in the enrichment analysis plot (Figures 2A,B). KEGG pathway analysis revealed 142 enriched signaling pathways. We generated an importance bubble plot and a classification histogram (Figures 2C,D) to visually represent the top 20 KEGG pathways, ranked in ascending order according to FDR values and corroborated by prior research.

The GO and KEGG analyses demonstrated that these genes are widely distributed and expressed across various subcellular localizations. Notably, many of the identified genes are involved in key regulatory processes, including neuronal transmission, cell proliferation, cell cycle control, apoptosis, and signal transduction. Among the KEGG pathway enrichments, several pathways stood out, including the PI3K-AKT signaling pathway, lipid and atherosclerosis, and the HIF-1 signaling pathway. These findings suggest that the therapeutic efficacy of CEP in treating BPH may be mediated through multiple biological processes and signaling pathways, thereby providing insights into its underlying molecular mechanisms.

#### 3.4.2 Pathway enrichment analysis of core targets

To elucidate the biological pathways associated with the 20 central targets of CEP in the treatment of BPH, we conducted a comprehensive KEGG pathway enrichment analysis using the DAVID, FUMA, and Metascape databases. Among the 142 significant signaling pathways identified, we constructed an enriched KEGG term network (Figure 2E) and categorized these pathways into distinct functional clusters based on their biological relevance. Additionally, we generated a Sankey diagram (Figure 2F) that integrates the FDR values with the top 20 enriched pathways, ranked according to relevant studies and literature, while highlighting overlapping genes among these pathways.

Our analysis revealed that the primary pathways associated with the central targets of CEP in the treatment of BPH are closely related to the PI3K-AKT signaling pathway, EGFR tyrosine kinase inhibitor resistance, MAPK signaling pathway, HIF-1 signaling pathway, and mTOR signaling pathway. Furthermore, the central targets were significantly enriched in pathways involved in apoptosis, signal transduction, hormone response, and oxidative stress.

### 3.5 Molecular docking for CEP with core target of BPH

To further elucidate the interactions and potential mechanisms between CEP and four key targets (AKT1, EGFR, SRC, and FN1) in the treatment of BPH, we performed comprehensive molecular docking simulations. Using the CB-Dock online tool, we generated docking models for each target. Notably, all models exhibited binding energies lower than −5.0 kcal/mol, indicating a strong binding affinity between CEP and these targets. This suggests that CEP can spontaneously bind to these core targets, playing a significant role in its molecular mechanism for treating BPH. Detailed VINA scores and docking energies are provided in Table 2.

**TABLE 2:** Molecular docking CurPocket.

Name	UniProtKB	PDB	CurPock ID	Vina score	Cavity volume	Center (x,y,z)	Docking size (x,y,z)
AKT1	P31749	1H10	C5	−7.7	96	15, 21, 16	23, 23, 23
			C4	−7.5	102	16, 16, −2	23, 23, 23
			C1	−7.3	160	28, 24, 7	23, 23, 23
			C3	−7.1	110	17, 5, 14	23, 23, 23
			C2	−6.3	152	23, 19, 22	23, 23, 23
EGFR	P00533	1M14	C1	−9.2	3283	29, 9, 50	32, 35, 23
			C4	−7.5	172	17, 27, 52	23, 23, 23
			C2	−7.2	453	38, 20, 67	23, 23, 23
			C3	−7.1	231	31, −5, 63	23, 23, 23
			C5	−6.7	169	22, −7, 61	23, 23, 23
SRC	P12931	1A07	C4	−7.5	96	53, 10, 35	23, 23, 23
			C3	−7.2	145	34, 5, 35	23, 23, 23
			C1	−7.0	1209	42, 12, 28	23, 23, 23
			C5	−7.0	82	40, −5, 36	23, 23, 23
			C2	−6.5	171	43, 10, 14	23, 23, 23
FN1	P02751	4GH7	C4	−9.6	1289	9, −72, −23	23, 23, 23
			C1	−9.2	9024	51, −66, −23	35, 33, 23
			C3	−8.9	3710	25, −59, −25	23, 29, 23
			C2	−8.2	5909	−3, −53, −30	34, 23, 29
			C5	−7.9	1044	34, −82, −33	23, 23, 23

**FIGURE 3:**
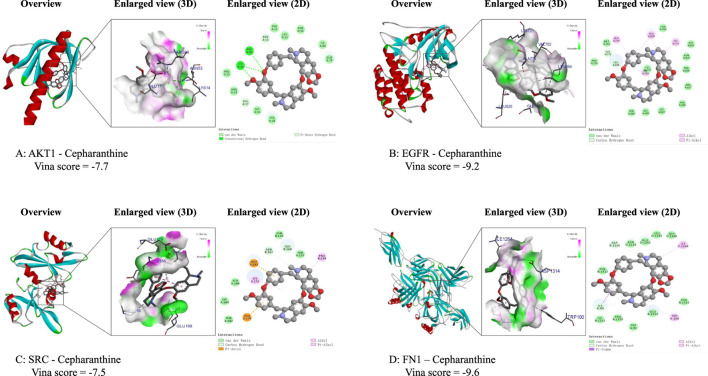
Molecular docking results of the lowest binding energy in each target with CEP. **(A)** The 2D force and 3D spatial environment between CEP and AKT1; **(B)** The 2D force and 3D spatial environment between CEP and EGFR; **(C)** The 2D force and 3D spatial environment between CEP and SRC; **(D)** The 2D force and 3D spatial environment between CEP and FN1.

We utilized Discovery Studio software to visualize the lowest energy binding conformations between CEP and each key target, generating both 2D and 3D representations (Figure 3). These visualizations provided insights into the molecular interactions and binding modes between CEP and the target proteins, highlighting their potential roles in mediating the drug’s therapeutic effects.

### 3.6 Calculation of IC_50_ of CEP inhibition of WPMY-1 and BPH-1

**FIGURE 4:**
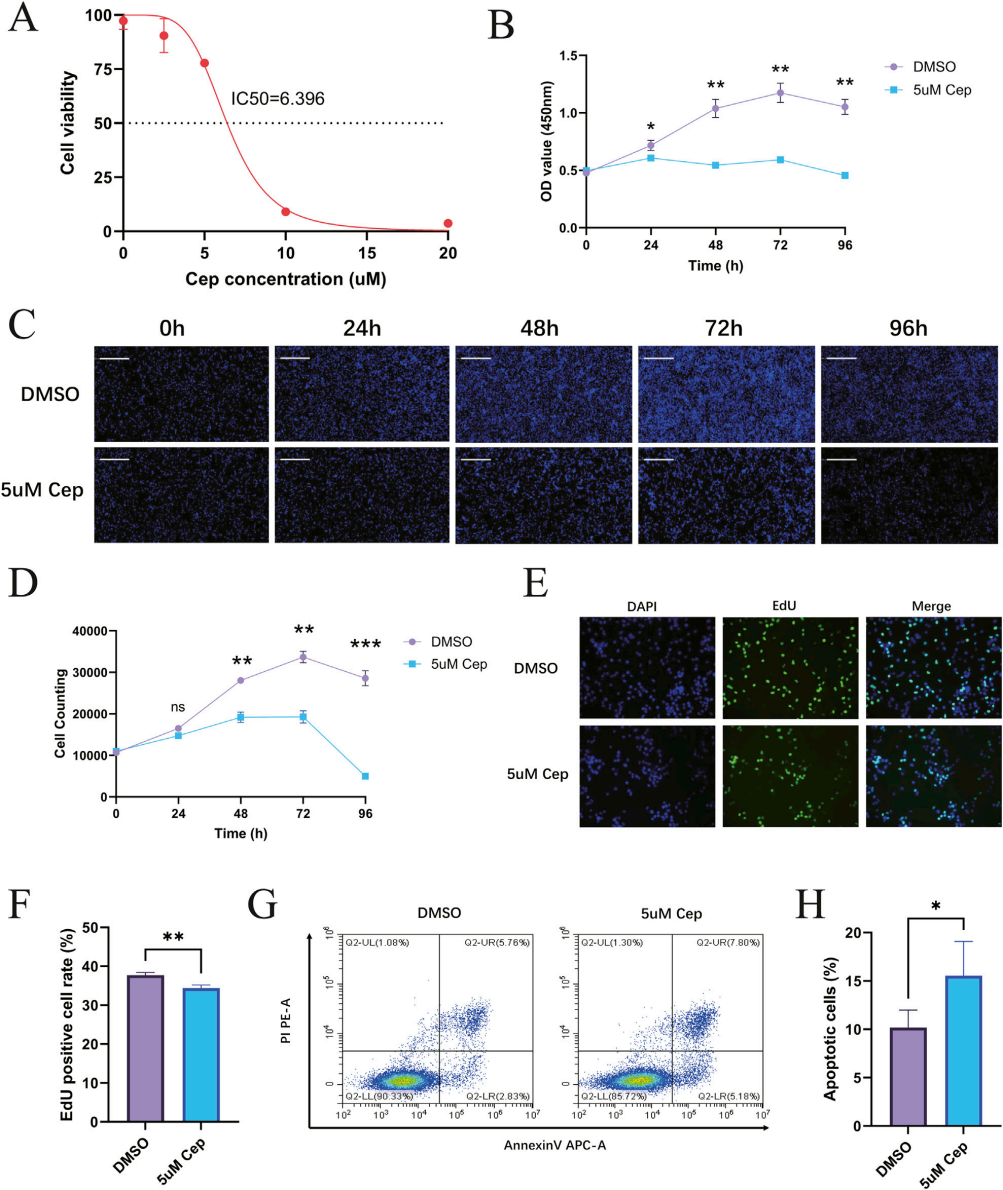
CEP can inhibit the proliferation of WPMY-1 *in vitro* and promote the apoptosis of WPMY-1. **(A)** The dose-effect curve fitted by four-parameter nonlinear regression showed that the drug concentration corresponding to a 50% inhibition rate was IC_50_ = 6.396 μM; **(B)** CCK-8 found that 5 μM CEP inhibited WPMY-1 cell proliferation in a time-dependent manner; **(C)** Celigo full-view cell scanning analyzer also confirmed that 5 μM CEP showed a significant time-dependent inhibition of WPMY-1 cell proliferation. Scale bar = 500 μm; **(D)** WPMY-1 cell proliferation curve (0–96 h) based on the results of Celigo full-view cell scanning analyzer; **(E)** EdU staining assay visually showed that CEP significantly inhibited the proliferation of WPMY-1 cells. Scale bar = 100 μm; **(F)** Quantitative analysis of EdU staining; **(G)** Annexin V-APC/PI double staining flow cytometry showed that 5 μM CEP treatment for 48 h significantly induced apoptosis of WPMY-1 cells; **(H)** Quantitative analysis of apoptosis detection. Data are presented as mean ± SD, and were analyzed with One-way ANOVA with Tukey’s post-hoc test. *p < 0.05, **p < 0.01, ***p < 0.001. NS, Non-significant; Cep, Cepharanthine.

CCK-8 assay showed that CEP exhibited a dose-dependent inhibition on the viability of WPMY-1 cells in the concentration range of 0–20 μM. As the concentration of CEP increased, the cell viability decreased in a step-like manner. The dose-effect curve fitted by four-parameter nonlinear regression showed that the drug concentration corresponding to a 50% inhibition rate was IC_50_ = 6.396 μM (95%CI 5.915–6.912 μM) (Figure 4A). Based on this, 5 μM was selected for treatment in subsequent experiments: this concentration maintained the viability at 65%–70% (corresponding to a 35%–40% inhibition rate), which can effectively observe the drug effect and avoid the cytotoxic interference caused by high concentrations, thus ensuring the reliability of mechanism research. In addition, CCK-8 assay showed that CEP exhibited a dose-dependent inhibition on the viability of BPH-1 cells in the concentration range of 0–20 μM. As the concentration of CEP increased, the cell viability decreased in a step-like manner. The dose-effect curve fitted by four-parameter nonlinear regression showed that the drug concentration corresponding to a 50% inhibition rate was IC_50_ = 2.355 μM (95%CI 2.103–2.632 μM) (Supplementary Appendix 3A). Based on this, 2.5 μM was selected for treatment in subsequent experiments.

### 3.7 CCK-8 and celigo live cell counting assays to detect the inhibitory effect of CEP on WPMY-1 and BPH-1 proliferation

WPMY-1 cells were divided into a DMSO control group and a 5 μM CEP treatment group. CCK-8 was used to detect cell proliferation activity at five time points: 0, 24, 48, 72, and 96 h. The results showed that 5 μM CEP inhibited WPMY-1 cell proliferation in a time-dependent manner. The cell activity of the DMSO control group continued to rise, reaching a peak at 72 h, and slightly declined at 96 h due to contact inhibition; the activity of the CEP treatment group was always significantly lower than that of the control group, indicating that CEP can effectively block the cell proliferation process of WPMY-1 (Figure 4B). In addition, non-labeled live cell counting was performed using the Celigo full-view cell scanning analyzer. The results also confirmed that 5 μM CEP showed a significant time-dependent inhibition of WPMY-1 cell proliferation: the number of cells in the DMSO control group continued to rise, reaching a peak at 72 h, and slightly declined at 96 h due to contact inhibition; the proliferation of the CEP treatment group was not significantly affected in the early stage (24 h), growth stagnation began at 48 h, and severe cell disintegration occurred at 72–96 h. Morphological analysis showed that the cell density in the CEP group dropped sharply after 48 h, and the widespread signal disappeared after 96 h, confirming that CEP may inhibit the cell activity of WPMY-1 through a two-stage mechanism of first blocking proliferation and then inducing cell death (Figures 4C,D).

Furthermore, BPH-1 cells were divided into a DMSO control group and a 2.5 μM CEP-treated group. Cell proliferation activity was assessed using the CCK-8 assay at five time points: 0, 24, 48, 72, and 96 h. Results showed that 2.5 μM CEP exhibited a time-dependent inhibitory effect on BPH-1 cell proliferation. Cell viability in the DMSO control group continued to increase, reaching a peak at 72 h, and then decreased significantly at 96 h due to contact inhibition. Cell viability in the CEP-treated group remained significantly lower than that in the control group, demonstrating that CEP effectively blocked BPH-1 cell proliferation (Supplementary Appendix 3B). Label-free viable cell counts were performed using a Celigo full-field cell analyzer. Results also confirmed that 2.5 μM CEP exhibited a significant time-dependent inhibitory effect on BPH-1 cell proliferation: Cell number in the DMSO control group continued to increase, reaching a peak at 72 h, and then decreased slightly at 96 h due to contact inhibition. In the CEP-treated group, cell proliferation showed no significant effect early in the 24-h period, but began to arrest at 48 h, and severe cell disintegration occurred between 72 and 96 h (Supplementary Appendix 3C).

### 3.8 EdU staining to detect the inhibitory effect of CEP on WPMY-1 and BPH-1 proliferation

EdU staining assay visually showed that CEP significantly inhibited the proliferation of WPMY-1 cells. In the DMSO control group, a large amount of EdU^+^ green signals were distributed in the DAPI^+^ blue nuclei, indicating that the cells were actively proliferating; the proportion of EdU^+^ cells in the CEP treatment group decreased sharply, and the green signals were sparsely distributed in a dot-like manner. Merge further confirmed that the cell density in the CEP group decreased and nuclear fragmentation increased, verifying the inhibitory effect of CEP on WPMY-1 cell proliferation from the perspective of DNA synthesis (Figures 4E,F).

Similarly, EdU staining experiments visually demonstrated that CEP also significantly inhibited BPH-1 cell proliferation. In the DMSO control group, abundant EdU^+^ green signals were distributed within DAPI^+^ blue nuclei, indicating active cell proliferation. In the CEP-treated group, the proportion of EdU cells decreased dramatically, and the green signals were sparsely distributed in a punctate pattern. Merge experiments further confirmed the decreased cell density and increased nuclear fragmentation in the CEP group, confirming CEP’s inhibitory effect on BPH-1 cell proliferation from the perspective of DNA synthesis (Supplementary Appendix 3E).

### 3.9 CEP can promote apoptosis of WPMY-1 cells and BPH-1 cells

Annexin V-APC/PI double staining flow cytometry showed that 5 μM CEP treatment for 48 h significantly induced apoptosis of WPMY-1 cells. The proportion of late apoptotic cells (Annexin V^+^/PI^+^, upper right quadrant) in the CEP group increased significantly, while the proportion of early apoptotic cells (Annexin V^+^/PI^−^, lower right quadrant) also increased (Figures 4G,H). The characteristic distribution of apoptotic cells clearly migrated toward the Annexin V^+^ quadrant, suggesting that CEP inhibits the activity of prostate stromal cells by dually promoting early and late apoptosis processes.

Annexin V-FITC/PI double staining combined with flow cytometry was used to examine the inhibitory effect of CEP on apoptosis in BPH-1 cells. As shown in Supplementary Appendix 3D, after 48 h of treatment with 2.5 μM CEP, the proportion of apoptotic cells was significantly altered compared to the DMSO control group. The proportion of early apoptotic cells (Annexin V^+^/PI^−^) in the CEP-treated group was significantly increased, while the proportion of late apoptotic and necrotic cells (Annexin V^+^/PI^+^) also increased to a certain extent. These results indicate that 2.5 μM CEP treatment effectively induces apoptosis in BPH-1 cells, primarily by significantly increasing the proportion of early apoptotic cells.

### 3.10 Western blot confirmed that CEP can inhibit the proliferation of WPMY-1 and BPH-1 by inhibiting the EGFR/PI3K/AKT signaling pathway

**FIGURE 5:**
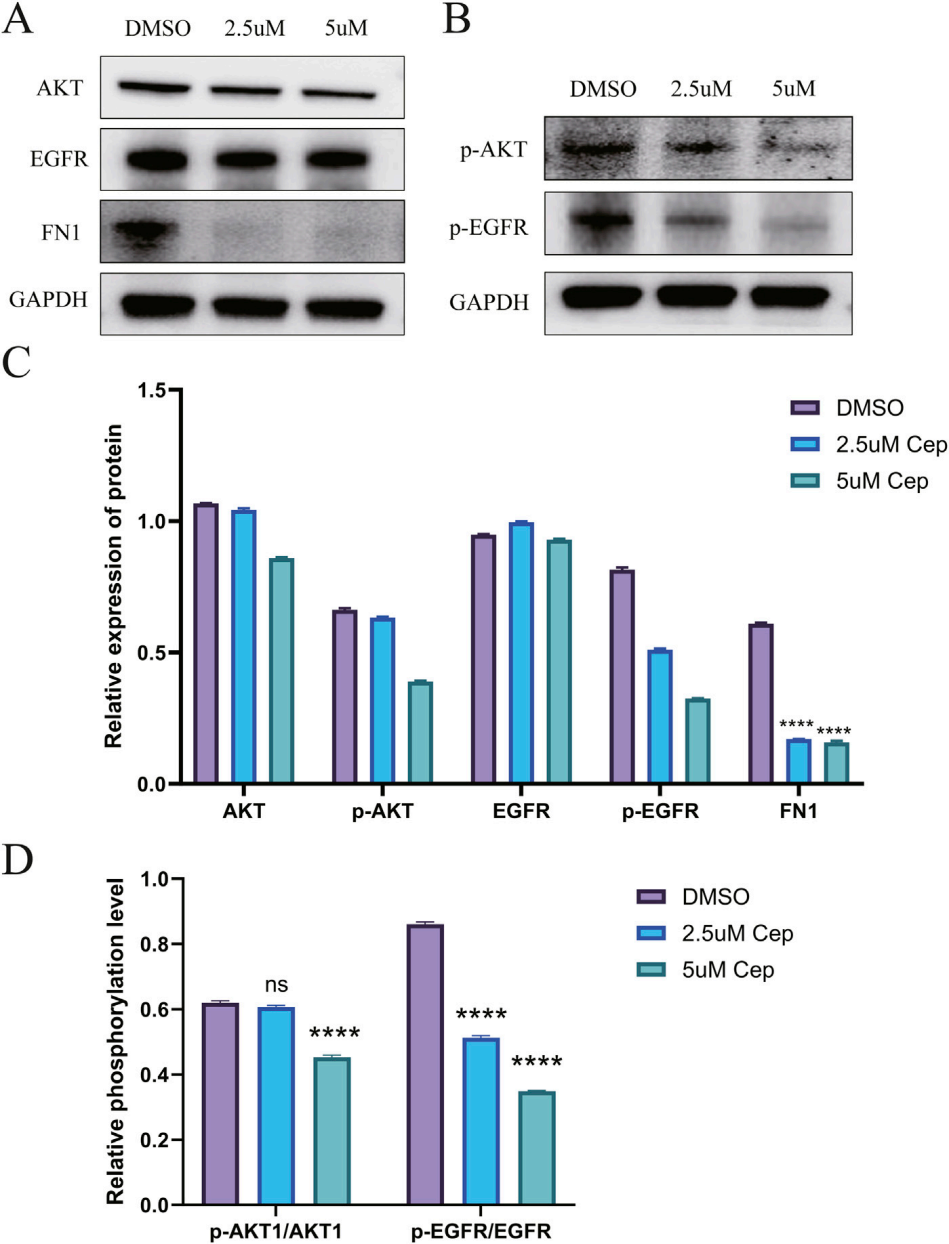
Western blot confirmed that CEP can inhibit the proliferation of WPMY-1 by inhibiting the EGFR/PI3K/AKT signaling pathway. **(A)** Western blot analysis showed that CEP could significantly inhibit the expression of FN1 and also had a certain inhibitory effect on the total protein expression of AKT and EGFR, but there was no statistical difference; **(B)** Compared with the DMSO group, 2.5 μM CEP treatment for 48 h significantly reduced the expression of p-AKT and p-EGFR, and 5 μM CEP further aggravated the inhibitory effect; **(C)** Quantitative analysis of AKT, p-AKT, EGFR, p-EGFR and FN1 by Western blot; **(D)** phosphorylation level analysis simultaneously showed that the ratios of p-AKT/AKT and p-EGFR/EGFR in the CEP group were significantly decreased. Data are presented as mean ± SD, and were analyzed with One-way ANOVA with Tukey’s post-hoc test. ****p < 0.0001. NS: Non-significant; Cep: Cepharanthine.

For WPMY-1, Western blot analysis showed that CEP inhibited the activation of the EGFR/PI3K/AKT signaling pathway and the expression of FN1 in a concentration-dependent manner. Compared with the DMSO group, 2.5 μM CEP treatment for 48 h significantly reduced the expression of p-AKT and p-EGFR, and 5 μM CEP further aggravated the inhibitory effect; phosphorylation level analysis simultaneously showed that the ratios of p-AKT/AKT and p-EGFR/EGFR in the CEP group were significantly decreased. The expression of the downstream effector protein FN1 decreased in a step-by-step manner with the increase in CEP concentration, confirming that CEP inhibited the synthesis of FN1, a key component of the extracellular matrix, by blocking the EGFR/PI3K/AKT signaling cascade, thereby inhibiting the proliferation of WPMY-1 cells (Figure 5).

Results from the BPH-1 cell line showed that, compared with the DMSO control group, CEP treatment for 48 h at 2.5 μM and 5 μM decreased the expression of the extracellular matrix protein FN1 in a concentration-dependent manner. Importantly, while the total protein levels of key signaling pathway proteins EGFR and AKT did not change significantly, their phosphorylation levels (p-EGFR and p-AKT) decreased significantly with increasing CEP concentration, indicating that CEP can effectively inhibit activation of the EGFR/AKT signaling pathway. These results suggest that CEP may exert its inhibitory effect on cell viability by inhibiting the EGFR/AKT signaling pathway, thereby affecting the expression of downstream functional proteins such as FN1 (Supplementary Appendix 4).

## 4 Discussion

Current clinical treatments for BPH primarily rely on pharmacological interventions. α1-adrenergic receptor blockers alleviate symptoms by relaxing prostate smooth muscle but can easily induce orthostatic hypotension (Papet et al., 2025). 5α-reductase inhibitors, while capable of shrinking the prostate, are associated with side effects such as decreased libido and erectile dysfunction (Soda et al., 2025). CEP, a multi-target natural compound, exerts anti-proliferative and pro-apoptotic effects through dual inhibition of EGFR and AKT, while also possessing a favorable safety profile. It holds promise as a novel, highly effective, and low-side-effect targeted therapy.

This study is the first to systematically analyze the molecular mechanism of the natural alkaloid CEP in the targeted regulation of BPH through a multi-dimensional integration strategy. Experimental data showed that CEP effectively inhibited the proliferation activity of human prostate stromal cells WPMY-1 and BPH-1 with a low micromolar IC_50_ value. Dynamic proliferation monitoring revealed that CEP presented a unique “temporal and spatial dual-stage regulation mode”: Based on the findings of this study and previous studies, we speculate that in the early intervention stage (24–48 h), CEP may significantly inhibit DNA synthase activity by blocking the G1/S phase transition of the cell cycle (Payon et al., 2019); in the late effect stage (72–96 h), CEP may induce the apoptosis pathway of WPMY-1 and BPH-1, causing cytoskeleton disintegration and membrane permeability changes (Xu et al., 2020; Liang et al., 2023). This dynamic process from proliferation blockade to apoptosis induction has important clinical significance in the treatment of BPH - early rapid relief of urethral mechanical obstruction, and long-term fundamental reversal of the pathological process of matrix remodeling, providing a dual guarantee mechanism for the translational application of CEP. Similar to the findings of this study, studies have reported that CEP also has a significant inhibitory effect on prostate cancer cells (Dong et al., 2025). The latest *in vitro* experiments by Dong et al. showed that CEP inhibited the proliferation and migration of prostate cancer cells (PC-3 and DU145) in a concentration-dependent manner and induced apoptosis, and knockout or drug inhibition of DUSP1 could partially reverse the anti-cancer effect of CEP, confirming that DUSP1 is its key mediator. At the same time, the *in vivo* mouse transplant tumor model further verified that CEP can significantly inhibit tumor growth by upregulating DUSP1 in tumor tissues and reducing the level of phosphorylated ERK (Dong et al., 2025).

The network analysis results of this study showed that the anti-BPH effect of CEP was related to four core targets: AKT1, EGFR, SRC and FN1. The docking energy of CEP with EGFR was −9.2 kcal/mol, the docking energy of AKT1 was −7.7 kcal/mol, the docking energy of FN1 was −9.6 kcal/mol, and the docking energy of SRC was −7.5 kcal/mol. Among them, EGFR, AKT1 and FN1 are key genes in the EGFR/PI3K/AKT signaling pathway. Therefore, we performed Western blot verification in WPMY-1 and BPH-1 cells. The results showed that after 48 h of treatment with 5 μM CEP, the expression of phosphorylated EGFR (Tyr1068) and phosphorylated AKT (Ser473) was significantly reduced, and the expression of the downstream effector protein FN1 was also significantly inhibited, and the inhibitory effect was strictly concentration-dependent. Based on the above findings, we speculate that the EGFR/PI3K/AKT signaling axis is the core target of CEP to exert its anti-proliferative effect. This dual high-affinity inhibition of EGFR and AKT explains at the atomic level the molecular basis of CEP’s efficient blocking of signaling pathways at micromolar concentrations. Similar to this study, this effect of CEP has also been verified in tumors (Yang et al., 2024). Yang et al. found that CEP significantly inhibited the proliferation and cloning ability of nasopharyngeal carcinoma cells in a dose-dependent manner (Yang et al., 2024). The results of their network analysis experiments showed that the anti-nasopharyngeal carcinoma effect of CEP was related to eight core targets such as EGFR, AKT1, PIK3CA and mTOR. By performing molecular docking, the binding ability of CEP with candidate core proteins (EGFR, AKT1, PIK3CA and mTOR) was predicted, and the docking energy of EGFR was −10.0 kcal/mol, PIK3CA was −12.4 kcal/mol, AKT1 was −10.8 kcal/mol, and mTOR was −8.6 kcal/mol. Western blot analysis showed that CEP effectively inhibited the expression of EGFR and the phosphorylation levels of downstream signaling proteins (including PI3K, AKT, mTOR and ERK) (Yang et al., 2024).

It is particularly noteworthy that FN1, as a core regulator of extracellular matrix remodeling, has profound pathophysiological significance in its downregulation. FN1 is an important extracellular matrix glycoprotein that participates in physiological and pathological processes (Wang et al., 2022; Xiang et al., 2022; Xu H. et al., 2024; Zhou et al., 2024). Studies have reported that FN1 can stimulate the proliferation of growth-arrested mammary epithelial cells, induce EMT response, disrupt the cavitary acinar structure, and promote tumor-like behavior (Park and Schwarzbauer, 2014; Konac et al., 2017). At the same time, FN1 may play a key role in fibrosis (Cardoso et al., 2018). In the progression of BPH, we speculate that FN1 may promote collagen I/III deposition and increase matrix hardness, while forming a pro-fibrotic positive feedback through an autocrine loop. This study found that CEP significantly inhibited the expression of FN1 by blocking the EGFR/PI3K/AKT signaling pathway. The role of FN1 in the occurrence and development of BPH may have potential relevance to the progression of prostate cancer. Treacy et al. conducted a retrospective analysis of 695 patients with localized prostate cancer who underwent radical prostatectomy and received Decipher transcriptome testing. Their gene selection chip analysis showed that FN1 was significantly overexpressed in patients with extra-capsular extension and lymph node invasion (Treacy et al., 2023).

The “computational prediction-experimental verification” paradigm successfully implemented in this study demonstrates the unique value of multi-omics integration strategy in the analysis of natural drug mechanisms. Network analysis screened 96 cross-targets from 142 CEP targets and 5,081 BPH-related targets. The constructed PPI network showed that the core targets included EGFR, AKT1, FN1 and SRC, confirming their signaling hub status. Among the top 20 pathways in KEGG enrichment analysis, the PI3K-AKT pathway was experimentally confirmed as the main target, while the HIF-1 and MAPK pathways were not verified in this study, but they had significant cross-reactions with PI3K-AKT. *In vitro* studies of prostate cancer have shown that AKT, as an upstream regulatory hub, can directly stabilize and activate the expression and function of HIF-1α through the PI3K/AKT signaling pathway (Lee et al., 2022). Lee et al. found that in the hypoxic tumor microenvironment, the continuous activation of AKT leads to the accumulation of HIF-1α protein in PTEN-deficient prostate cancer cells, which in turn drives the expression of its downstream target genes, promotes angiogenesis, tumor invasion and the progression of castration-resistant prostate cancer (CRPC); at the same time, the overexpression of HIF-1α will further aggravate the abnormal activation of the androgen receptor (AR) signaling pathway, forming an AKT-HIF-1α-AR positive feedback loop, which jointly mediates cancer cell proliferation, migration, apoptosis resistance and treatment failure (Lee et al., 2022). In addition, some studies have reported that AKT can negatively regulate MAPK signaling by phosphorylating RAF, while ERK can activate mTORC1 by phosphorylating TSC2, forming a bidirectional regulation. In the treatment of prostate cancer resistance, inhibition of AKT/mTOR will feedback enhance MAPK signaling, and conversely, MEK inhibitors will relieve S6K’s inhibition of IRS1, activate the PI3K-AKT pathway, and lead to compensatory escape. This dynamic interaction confirms that co-targeting MAPK and AKT can synergistically inhibit prostate cancer growth and delay the progression of castration resistance (Shorning et al., 2020). The above pathway interaction network suggests that CEP may play a role through a multidimensional mechanism of “deep inhibition of the main target and coordinated regulation of the bypass pathway”. In this study, the strict correspondence between the molecular docking results and the experimental data not only verified the reliability of the computational model, but also provided theoretical guidance for experimental design.

Compared with existing BPH therapies, CEP is expected to show potential therapeutic advantages in three dimensions: First, the targeting specificity is improved. Traditional 5α-reductase inhibitors mainly act on androgen signals in epithelial cells (Madersbacher et al., 2019), but have limited intervention on matrix proliferation (Vickman et al., 2020). This study found that CEP has the potential to selectively inhibit the EGFR/PI3K/AKT pathway of stromal cells, which is expected to match the pathological characteristics of BPH dominated by matrix proliferation (Xu G. et al., 2024). Second, CEP has a multi-pathway synergistic inhibition of BPH mechanism, which is expected to overcome BPH resistance. This study found that CEP may simultaneously regulate proliferation, apoptosis and fibrosis, achieving a transition from symptom relief to pathological reversal. Finally, the safety of CEP oral treatment is guaranteed. The latest clinical trials have shown that CEP has good safety in treating asymptomatic or mild COVID-19 patients (Wei et al., 2025). In a double-blind randomized controlled trial (NCT05398705), patients received 120 mg/day, 60 mg/day CEP or placebo for 5 days. The results showed that the incidence of adverse events in the three groups was similar (33.65% in the 120 mg group, 37.72% in the 60 mg group, and 35.35% in the placebo group). The most common adverse reactions were diarrhea (up to 12.5%), somnolence (8.77%), and night sweats (7.89%), all of which were mild to moderate (grade 1-2). No serious adverse events or deaths were reported, and the incidence of drug-related adverse events was not significantly different from that in the placebo group (23.68% in the 60 mg group vs. 20.20% in the placebo group). All adverse events were alleviated at the time of analysis, indicating that CEP is well tolerated in short-term treatment and has no major safety concerns (Wei et al., 2025).

The limitations of this study are mainly due to the phase attributes and resource focus of exploratory research: First, although the HIF-1/MAPK pathways predicted by network analysis were significantly enriched, experimental resources needed to be concentrated on verifying the core signal axis (EGFR/PI3K/AKT/FN1), because this pathway has the highest node degree in the PPI network and is directly associated with the proliferation/apoptosis phenotype, so other pathways have not been expanded for verification. Second, the lack of *in vivo* animal experiments is because CEP treatment of BPH is a new mechanism exploration, and the molecular target and effective concentration must be clarified through *in vitro* models to provide a basis for subsequent animal dose design. This “mechanism first” strategy is in line with the logic of drug development, but it needs to be supplemented with testosterone-induced BPH rat model verification in subsequent studies. Future research needs to be expanded in depth on the existing basis: First, the *in vivo* efficacy of CEP should be verified in the testosterone-induced BPH animal model, and its effects on prostate volume reduction rate, urodynamic parameters and tissue fibrosis degree should be evaluated by intraperitoneal injection. Secondly, it is necessary to establish a Transwell co-culture system of WPMY-1 stromal cells and BPH-1 epithelial cells to quantify the paracrine effects of CEP regulation and its inhibitory effect on epithelial hyperplasia in order to analyze the stromal-epithelial interaction mechanism. At the same time, experimental verification of key network enrichment pathways such as HIF-1 and MAPK should be supplemented to detect the effects of CEP on HIF-1α nuclear translocation and phosphorylated ERK levels under hypoxic microenvironment, and analyze its regulatory effect on oxidative stress markers. Although we used multiple databases for cross-validation, computational predictions carry inherent risks of false positives and false negatives. Network analysis can only provide potential mechanistic hypotheses, which require experimental validation to confirm their biological significance. Finally, a 3D organoid model can be developed based on primary cells of BPH patients, combined with Celigo dynamic scanning technology to track the spatial distribution and long-term antiproliferative effect of CEP in the glandular microenvironment, providing efficacy data closer to the physiological state for clinical transformation.

In summary, this study proposed a complete mechanism model for CEP in the treatment of BPH: CEP binds to the EGFR kinase domain and the PH domain of AKT with high affinity, blocks EGFR dimerization and PI3K membrane recruitment, inhibits AKT phosphorylation and reduces its translocation to the cell membrane. Inactivated AKT cannot activate downstream genes, ultimately inhibiting FN1 gene transcription. At the same time, experimental verification found that CEP can significantly promote the apoptosis of WPMY-1 and BPH-1 cells. This global regulation of the “proliferation-apoptosis-fibrosis” signaling network, coupled with CEP’s multi-target natural properties and good safety, makes it an ideal candidate drug for intervening in prostate stromal hyperplasia, providing the latest theoretical support for the development of new drugs for BPH. Although molecular docking results indicate that CEP has high binding affinity for targets such as EGFR and AKT1, these calculations represent only preliminary theoretical predictions. This study demonstrated that CEP significantly inhibited EGFR and AKT phosphorylation levels using Western blot analysis, providing experimental evidence for functional interactions between CEP and these targets. However, these results require further validation using more direct binding experiments (such as surface plasmon resonance or isothermal titration calorimetry) to accurately determine binding constants and specificity. Furthermore, its molecular targets and effective concentrations require further *in vitro* modeling to inform subsequent animal dose design. In summary, CEP’s multi-target potential, natural properties, and favorable safety profile make it an ideal candidate for the treatment of BPH. However, further *in vivo* pharmacokinetic and toxicology studies are needed to assess its clinical dosing window.

## 5 Conclusion

This study showed that the natural alkaloid CEP effectively blocked the proliferation of WPMY-1 and BPH-1 cells, and induced apoptosis by targeting the inhibition of the EGFR/PI3K/AKT signaling axis and downstream fibronectin FN1 expression. The multi-omics integration strategy confirmed that CEP exerted its therapeutic effect in a two-stage mode: early blockade of the cell cycle and late activation of the apoptotic pathway. This mechanism provides a molecular basis for CEP to treat benign prostatic hyperplasia. Its multi-target characteristics and good safety support its use as a candidate drug targeting BPH stromal hyperplasia, but further *in vivo* verification is needed to promote clinical translation.

## Data Availability

The datasets presented in this study can be found in online repositories. The names of the repository/repositories and accession number(s) can be found in the article/Supplementary Material.
