# Supplementary material for: Cepharanthine may inhibit the proliferation of prostate cells by blocking the EGFR/PI3K/AKT signaling pathway: comprehensive network analysis, molecular docking, and experimental evaluation
Source: Front Pharmacol. 2025 Nov 24;16:1654757. doi: 10.3389/fphar.2025.1654757 (PMC12682793; doi:10.3389/fphar.2025.1654757)
Supplement: Supplementary file 1 [file DataSheet4.pdf]

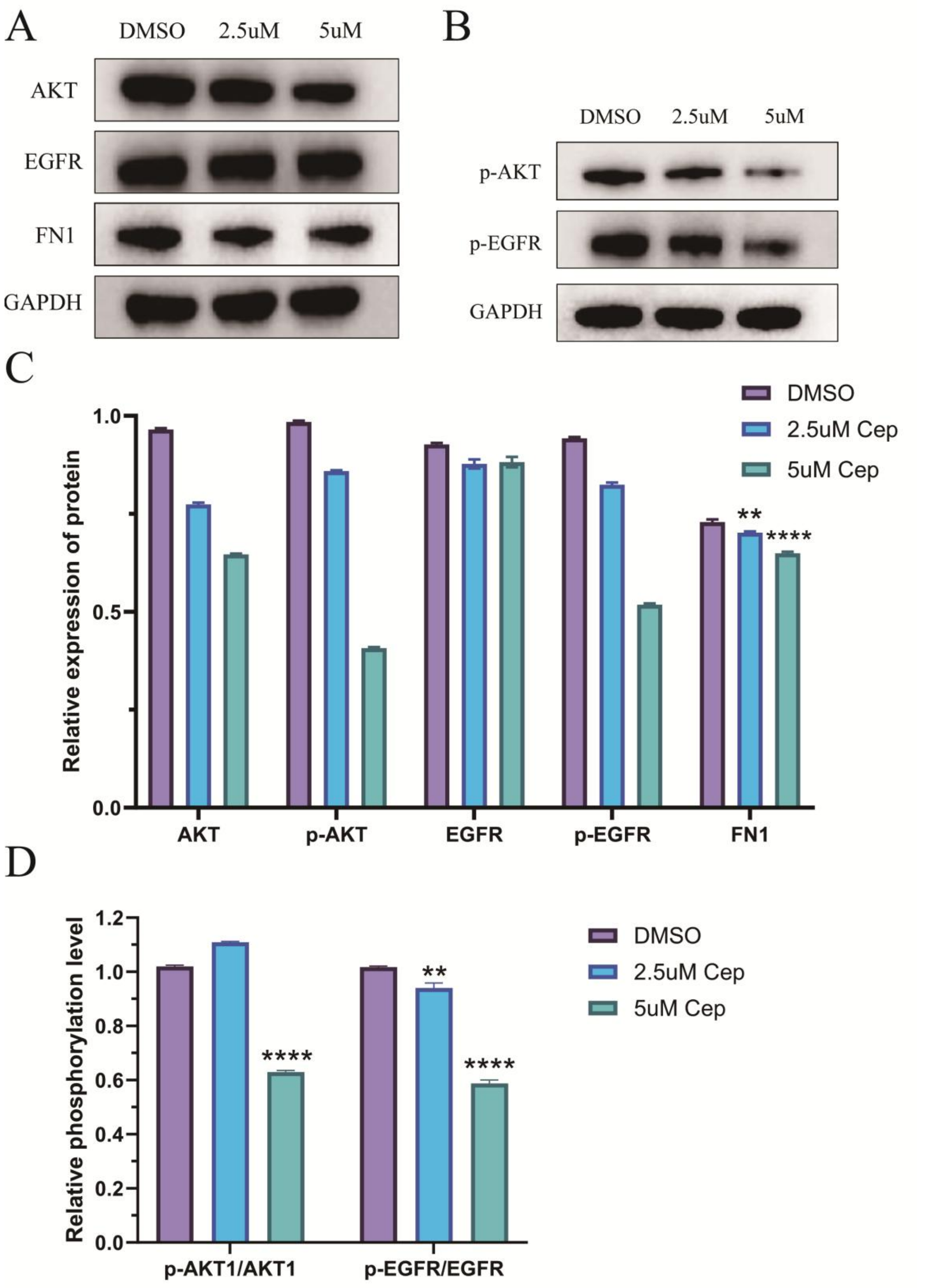

Appendix 4: Western blot confirmed that CEP can inhibit the proliferation of BPH-1 by inhibiting the EGFR/PI3K/AKT signaling pathway. (A): Western blot analysis showed that CEP could significantly inhibit the expression of FN1 and also had a certain inhibitory effect on the total protein expression of AKT and EGFR, but there was no statistical difference; (B): Compared with the DMSO group, 2.5 μM CEP treatment for

48 hours significantly reduced the expression of p-AKT and p-EGFR, and 5 μM CEP further aggravated the inhibitory effect; (C): Quantitative analysis of AKT, p-AKT, EGFR, p-EGFR and FN1 by Western blot; (D): phosphorylation level analysis simultaneously showed that the ratios of p-AKT/AKT and p-EGFR/EGFR in the CEP group were significantly decreased. Data are presented as mean ± SD, and were analyzed with One-way ANOVA with Tukey's post-hoc test. ****$p < 0.0001$. NS: Non-significant; Cep: Cepharanthine.
