# Supplementary material for: Cepharanthine may inhibit the proliferation of prostate cells by blocking the EGFR/PI3K/AKT signaling pathway: comprehensive network analysis, molecular docking, and experimental evaluation
Source: Front Pharmacol. 2025 Nov 24;16:1654757. doi: 10.3389/fphar.2025.1654757 (PMC12682793; doi:10.3389/fphar.2025.1654757)
Supplement: Supplementary file 2 [file DataSheet3.pdf]

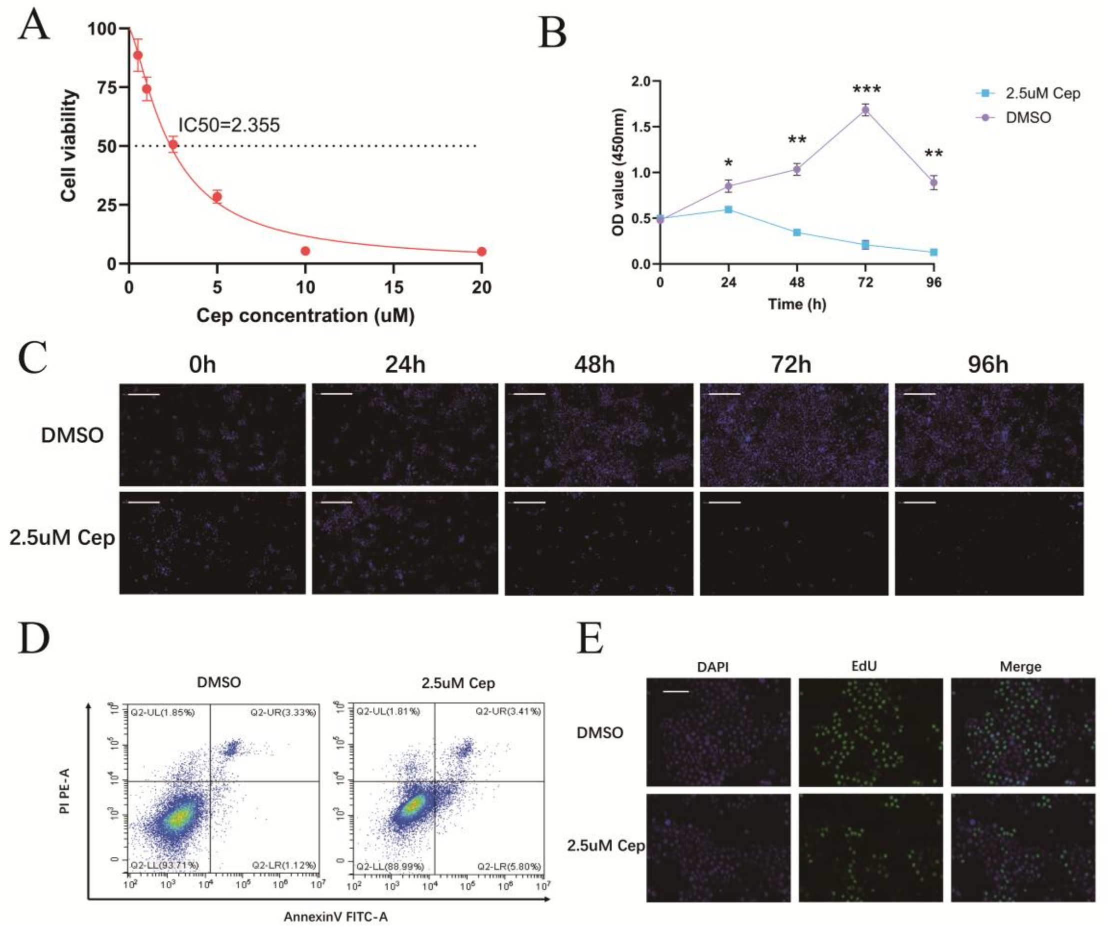

Appendix 3: CEP can inhibit the proliferation of BPH-1 in vitro and promote the apoptosis of BPH-1. (A): The dose-effect curve fitted by four-parameter nonlinear regression showed that the drug concentration corresponding to a 50% inhibition rate was IC_{50}=2.355 μM; (B): CCK-8 found that 2.5 μM CEP inhibited BPH-1 cell proliferation in a time-dependent manner; (C): Celigo full-view cell scanning analyzer also confirmed that 2.5 μM CEP showed a significant time-dependent inhibition of BPH-1 cell proliferation. Scale bar = 500μm; (D): Annexin V-FITC/PI double staining flow cytometry showed that 2.5 μM CEP treatment for 48 hours significantly induced apoptosis of BPH-1 cells; (E): EdU staining assay visually showed that CEP significantly inhibited the proliferation of BPH-1 cells. Scale bar = 100μm. Data are presented as mean ± SD, and were analyzed with One-way ANOVA with Tukey's post-hoc test. *$p < 0.05$, **$p < 0.01$, ***$p < 0.001$. NS: Non-significant; Cep: Cepharanthine.
