# Supplementary material for: Cepharanthine may inhibit the proliferation of prostate cells by blocking the EGFR/PI3K/AKT signaling pathway: comprehensive network analysis, molecular docking, and experimental evaluation
Source: Front Pharmacol. 2025 Nov 24;16:1654757. doi: 10.3389/fphar.2025.1654757 (PMC12682793; doi:10.3389/fphar.2025.1654757)
Supplement: Supplementary file 4 [file DataSheet1.pdf]

Data statement

We are grateful for the wealth of bioinformatics data provided by public databases. All data used in this study are sourced from various online platforms, including TCMIP (http://www.tcmip.cn/TCMIP/), SwissTargetPrediction (http://swisstargetprediction.ch/), GeneCards(http://www.genecards.org/), RCSB PDB (https://www.rcsb.org), DAVID (https://david.ncifcrf.gov/), FUMA (https://fuma.ctglab.nl/), among others. Detailed information on the databases and corresponding URLs can be found in Appendix. The raw data are available from the corresponding author upon reasonable request.

Steps	Works	Database/Tools	Website URL
1	Drug Efficacy Assessment	TCMIP	http://www.tcmip.cn/TCMIP/
2	Pharmaceutical Chemical Structure Retrieval	PubChem platform	https://pubchem.ncbi.nlm.nih.gov/
3	Drug Target Identification	Swisstargetprediction platform	http://www.swisstargetprediction.ch/
		CheMBL	https://www.ebi.ac.uk/chembl/
		SEA Search Server	https://sea.bkslab.org/
		STITCH	http://stitch.embl.de/
4	Disease-Associated Target Identification	GeneCards	http://www.genecards.org/
		TTD: Therapeutic Target Database	https://db.idrblab.net/ttd/
		OMIM: Online Mendelian Inheritance in Man	https://www.omim.org/
5	Drug-Disease Target Overlap Analysis	Venny 2.1	https://bioinfogp.cnb.csic.es/tools/venny/index.html
6	Protein Interaction Network Construction	STRING	https://STRINGdb.org/
		Cytoscape software 3.91	https://cytoscape.org
7	Functional Enrichment Analysis	DAVID	https://david.ncifcrf.gov/
		FUMA	https://fuma.ctglab.nl/
		WeiShengXin	https://www.bioinformatics.com.cn/
		Metascape	https://metascape.org/gp/index.html#/
8	Molecular Docking Simulation	Alphafold	https://alphafold.com/
		Uniprot	https://www.uniprot.org/
		RCSB PDB	https://www.rcsb.org
		CB-DOCK	https://cadd.labshare.cn/cb-dock2/
		Discovery-studio software	https://www.3ds.com/products/biovia/discovery-studio

Methodology Overview

To investigate potential drug interactions and mechanisms, we used a network pharmacology approach combined with molecular docking and various bioinformatics methods:

Step 1: Drug Efficacy Assessment

We collected active ingredients and efficacy data from the Traditional Chinese Medicine Integrated Pharmacology (TCMIP) database.

Step 2: Pharmaceutical Chemical Structure Retrieval

We used the PubChem platform to identify the SMILES representation and standard names of the active ingredients, ensuring accurate compound characterization.

Step 3: Drug Target Identification

Using the SMILES data, we predicted drug targets through several platforms: SwissTargetPrediction, ChEMBL, SEA Search Server, and STITCH. After merging and deduplication, we obtained a comprehensive set of drug targets.

Step 4: Disease-Associated Target Identification

We identified disease-related genes using GeneCards, the Therapeutic Target Database (TTD), and OMIM. The results were consolidated and validated through literature review.

Step 5: Drug-Disease Targets/ Venn Diagram

We used Venny 2.1 to create a Venn diagram showing the overlap between drug and disease targets, identifying candidate genes.

Step 6: Protein-Protein Interaction Network Construction

Protein interaction data for overlapping genes were collected from the STRING database and imported into Cytoscape to create a protein-protein interaction (PPI) network.

Step 7: Functional Enrichment Analysis

We used DAVID and FUMA databases to conduct Gene Ontology (GO) and KEGG pathway enrichment analyses, revealing key biological processes and pathways, and visualized in WeiShengxin and Metascape tools.

Step 8: Molecular Docking Simulation

Selected targets from the PPI network underwent molecular docking. Ligands were obtained from PubChem, and receptor structures were retrieved from Uniprot, PDB, and AlphaFold2. Docking was performed using CB-DOCK, and visualized in 2D/3D using Discovery Studio.
